# Does the Running Economy Really Increase after Ultra-Marathons?

**DOI:** 10.3389/fphys.2017.00783

**Published:** 2017-10-09

**Authors:** Gianluca Vernillo, Grégoire P. Millet, Guillaume Y. Millet

**Affiliations:** ^1^Human Performance Laboratory, Faculty of Kinesiology, University of Calgary, Calgary, AB, Canada; ^2^CeRiSM, Research Centre ‘Sport, Mountain and Health’, University of Verona, Rovereto, Italy; ^3^Institute of Sports Sciences, University of Lausanne, Lausanne, Switzerland

**Keywords:** energy cost, oxygen cost of human exercise, running, running economy, ultramarathon

## Overview

Over the years, ultra-endurance events have increasingly piqued the interest of the scientific community as they are considered an outstanding model to study the adaptive responses to both extreme loads and stresses on the human body (Millet and Millet, 2012). Notably, ultra-marathons, i.e., any event longer than the traditional marathon length of 42.195 km (Millet and Millet, 2012), have seen rising trends in participation (Hoffman et al., 2010; Cejka et al., 2014).

It is well-established that endurance running speed depends on the interaction between maximal oxygen uptake ($\dot{\text{V}}$O_2max_), the ability to sustain a high percentage of $\dot{\text{V}}$O_2max_ (fractional utilization of $\dot{\text{V}}$O_2max_), and a low economy of running (di Prampero et al., 1986). However, though the running economy (RE) is recognized to be a key determinant of running performance for “classic distances” (up to the marathon) (Saunders et al., 2004), whether or not it is also a primary determinant of ultra-marathon performances remains debated (Millet et al., 2011a, 2012b; Millet, 2012; Perrey et al., 2012). Indeed, although strategies to improve RE are mandatory in events shorter than or equal to the marathon distance, optimizing other factors associated with low-intensity endurance (e.g., minimizing damage to lower limb tissue and muscle fatigue) may cause the runners to choose strategies that lead to a deteriorated RE in ultra-marathons (Millet et al., 2012a,b). However, although $\dot{\text{V}}$O_2max_ and its fractional utilization have been described as determinants of ultra-marathon performances (Davies and Thompson, 1986; Millet et al., 2011a), it has been argued that the greatest variance in performance (~85%) was explained when the mean RE throughout was also added (Lazzer et al., 2012, 2014). Consequently, investigating the effects of fatigue on RE is still a crucial scientific question in ultra-marathon, both for performance optimization and a better understanding of the limits of the adaptive responses of the human body.

## The role of RE in the ultra-marathon

**Figure 1:**
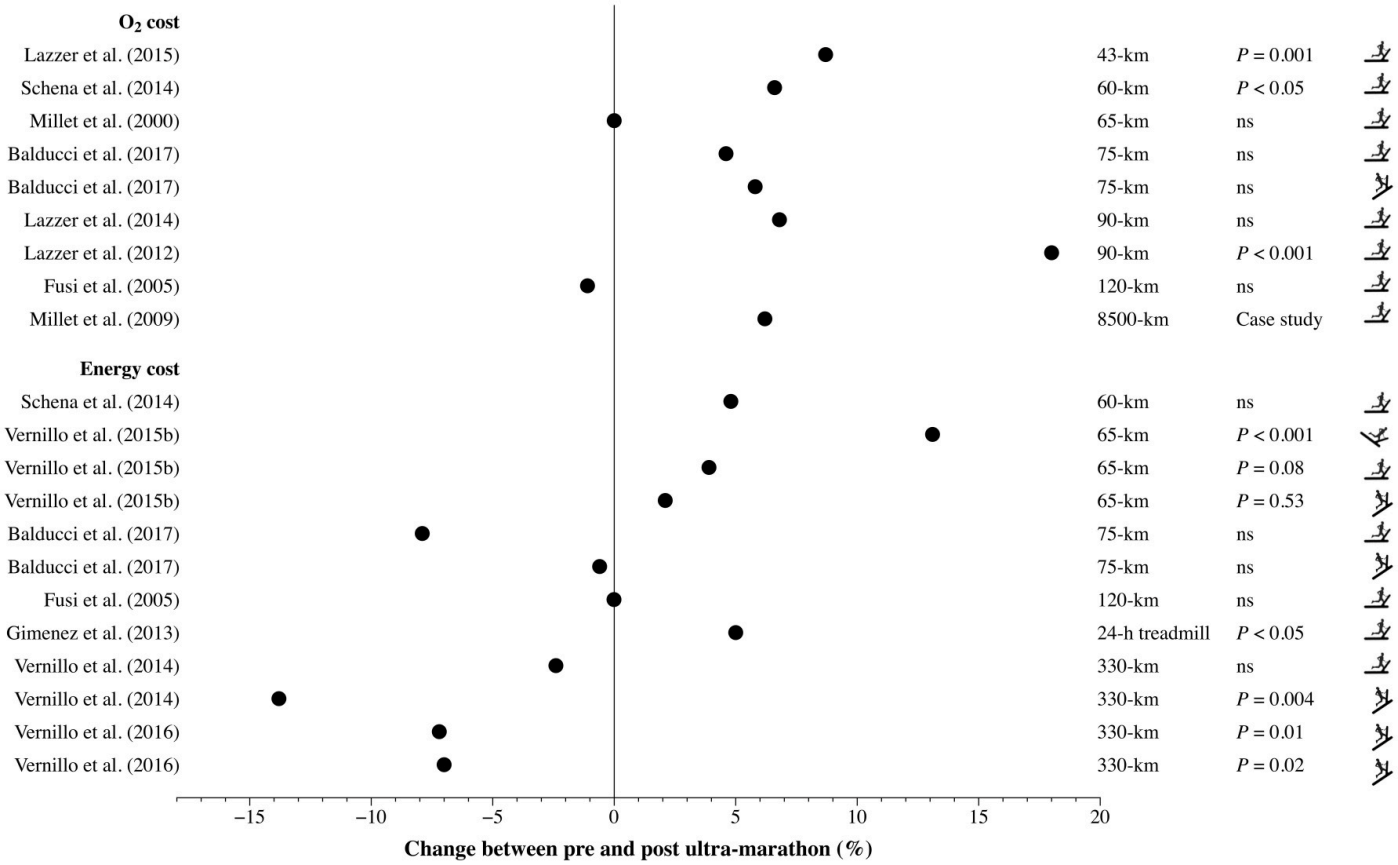
Summary of the studies examining the changes in running economy, either as O_2_ cost (mLO_2_·kg^−1^·m^−1^) or energy cost (J·kg^−1^·m^−1^), before (pre) and after (post) ultra-marathons. Ns denotes non-significant changes (*P* > 0.05) when the exact *P*-values were not available, whereas *P* < 0.05 and *P* < 0.001 indicate significant changes in cases where the exact *P*-values were not specified. Descendant, horizontal, or ascendant pictogram indicates that Cr has been assessed by means of a downhill, level, or uphill running protocol, respectively.

Two different forms of RE have been identified by the scientific literature; as oxygen cost or alternatively, as energy cost. By using one of the two approaches, many studies examined the effect of an ultra-marathon on RE, with equivocal findings (Figure 1). Manuscripts were acquired by searching the electronic databases of MEDLINE, PubMed, ScienceDirect, SPORTDiscus, and Web of Science using the following keywords in various combinations: “energy cost,” “oxygen cost,” “running economy,” “ultra-marathon,” “ultra-endurance.” We excluded articles written in languages other than English, as well as articles that have not yet been accepted or published. Electronic database searching was supplemented by examining the bibliographies of relevant articles.

### RE as oxygen cost

This approach involves the quantification of RE from the mass-specific $\dot{\text{V}}$O_2_, dividing the steady-state $\dot{\text{V}}$O_2_ above the value measured at rest in standing position, by the running speed. This is because $\dot{\text{V}}$O_2_ reflects the quantity of ATP used when the aerobic metabolism provides all of the energy (Fletcher et al., 2009). With this approach, referred to as O_2_ cost, a series of independent studies have reported increments by up to 18% after ultra-marathons lasting 60 to 53 km·day^−1^ for 161 days (Millet et al., 2009; Lazzer et al., 2012, 2015; Schena et al., 2014) (Figure 1, top panel). Surprisingly, not all the studies reported such increases (i.e., deteriorations) (Millet et al., 2000; Fusi et al., 2005; Lazzer et al., 2014).

### RE as energy cost

Since the energy yielded per liter of O_2_ depends on the substrate metabolized, and considering this later factor can vary with exercise intensity and/or duration, expressing RE in units of energy (i.e., the true energy cost of running, Cr) represents a better way to assess the energy used during running (Fletcher et al., 2009). Henceforth, Cr will therefore be used to express RE. It is our opinion that this is a crucial issue particularly within the ultra-marathons where there is a clear carbohydrate to fat shift from pre- to post-race (Davies and Thompson, 1986; Gimenez et al., 2013). Even so, when Cr was considered studies have still shown mixed results (Figure 1, bottom panel). Indeed, a study reported a ~13% increase in Cr after a 65-km mountain ultra-marathon (Vernillo et al., 2015b), whereas Gimenez et al. (2013) observed only a ~5% increase until the 8th h before level-Cr plateaued and remained fairly constant during a 24-h treadmill run. By contrast, other experiments showed no change (Fusi et al., 2005; Schena et al., 2014; Vernillo et al., 2014, 2015b; Balducci et al., 2017). Further, Vernillo et al. (2014) observed that Cr measured during uphill running decreased by ~14% after 330 km with a cumulative elevation gain of +24,000 m. The same authors confirmed and extended the previous observation (Vernillo et al., 2016), describing a ~7% decrease in Cr during different uphill running conditions after the same race.

Although the expression of RE as energy cost of running is an important issue in ultra-marathon (as highlighted above), the present opinion article will aim to address further physiological reasons and methodological issues that could potentially explain the presented discrepancies (Figure 1).

### What factors could explain an increased Cr after an ultra-marathon?

Cr has been reported to increase with the distance covered up to that of a marathon (Brueckner et al., 1991). Accordingly, an increased Cr after an ultra-marathon can also be expected, even though the mechanisms are not fully understood. Following ultra-marathons the functional capacity of the respiratory system can decrease (Vernillo et al., 2015a; Wüthrich et al., 2015). However, the cost of breathing at a given submaximal running speed is unclear. Indeed, $\dot{\text{V}}$O_2_ of the respiratory muscles (calculated from the pulmonary ventilation) was found to increase by ~18% (Millet et al., 2000; Vernillo et al., 2014); decreased by ~10% (Lazzer et al., 2015); or be unchanged (~3%) (Gimenez et al., 2013; Schena et al., 2014) after ultra-marathons from 60 to 330 km. Neuromuscular alterations may represent a source of Cr increase. Indeed, ultra-marathons lead to muscle fatigue and skeletal muscle damage (Martin et al., 2010; Millet et al., 2011b; Saugy et al., 2013) which needs to be compensated by a greater neural input to the muscle to produce the same amount of force, particularly during the push-off phase of the running step. This increased neural input to the muscle could cause a higher $\dot{\text{V}}$O_2_ demand (Bigland-Ritchie and Woods, 1974) and consequently a deteriorated Cr. Further, ultra-marathons can result in changes in running biomechanics pattern, particularly an increased stride frequency and leg stiffness (Morin et al., 2011; Degache et al., 2016). Most runners, in a non-fatigue state, spontaneously select a stride frequency that minimizes Cr (Cavanagh and Williams, 1982). However, whether or not this behavior persists in a fatigued state remains unclear. Finally, even though Achilles tendon stiffness remained similar after a marathon (Peltonen et al., 2012), a possible change in its mechanical properties cannot be ruled out after a longer mechanical loading such as during ultra-marathons. Accordingly, a potential decrease in the Achilles tendon stiffness would require a greater force generation during the push-off phase of the running step, leading to an elevated Cr (Roberts et al., 1998; Fletcher et al., 2010).

### What factors could explain that Cr does not increase after an ultra-marathon?

It must be acknowledged that an improved Cr after ultra-marathons found in some studies has been previously observed in other ultra-endurance tasks. Indeed, Cr of level walking has improved by ~19% after walking ~115 km·day^−1^ for 12 days (Tam et al., 2016), and gross efficiency has increased by ~15% after cycling 170 km·day^−1^ for 19 days (Slivka et al., 2012). Though the underlying mechanisms for these observations remain unclear, it is possible that ultra-endurance exercise induces positive adaptations in the neural control of the movement. Muscle fatigue can be reduced by a redistribution of the between-muscles activity level (Barry and Enoka, 2007), counteracting the reduced force production in the fatigued muscles (Turpin et al., 2011). The activation rotation between muscle or motor units is easier at low as compared to high intensity (Miller et al., 2012), which could represent another explanation for lower Cr deterioration in ultra-marathons.

### Methodological concerns

Several issues may be responsible for the discrepancies found in the literature regarding RE changes during ultra-marathons. First, the characteristics of the ultra-marathon (duration, continuous or stages race, course elevation, temperature, and altitude) make the determination of the sustained fraction of $\dot{\text{V}}$O_2max_ more challenging. It has been suggested that it could be as low as ~40–50% $\dot{\text{V}}$O_2max_ over a 24-h race (Millet et al., 2011a) and as high as ~70–80% $\dot{\text{V}}$O_2max_ over three running laps of 22, 48, and 20 km on 3 consecutive days (Lazzer et al., 2012). Second, despite the exponential rise in participation in ultra-marathons (Hoffman et al., 2010; Cejka et al., 2014), the number of finishers is currently less than 13% compared to the marathon (Medinger, 2015). Thus, the number of competitors remains limited and ultra-marathoners continue to be a relatively small subset of runners. These considerations make the analysis of the athletes' level of performance more challenging (Millet, 2012; Perrey et al., 2012). Thus, to what extent Cr changes can be elicited, and what mechanisms precipitate these changes remain open research questions. Yet, we believe that methodological limitations represent another candidate that might explain the above-mentioned discrepancies (Figure 1). For example, some studies during mountain ultra-marathons used level running protocols to analyze changes in Cr while graded running conditions should be a more accurate model to study this type of performance, mainly characterized by large positive/negative elevation changes (Vernillo et al., 2015b; Balducci et al., 2016). Additionally, the individual changes in Cr should be reported along with the mean and standard deviation. A range of individual responses may present the same mean and standard deviations (Weissgerber et al., 2015) and because ultra-marathons present unique and specific characteristics (see above) (Millet et al., 2012b), the variability in these events is expected to be higher than that found in shorter distances. Thus, the identification of individual responses is probably even more critical. Further, for the studies that use treadmill running to assess the changes in Cr, several sessions are required to familiarize the subjects with treadmill running (Brueckner et al., 1991) and more importantly, to the testing conditions themselves (e.g., slope of the treadmill). This has not always been done properly. Finally, though studies on changes in running mechanics (Degache et al., 2016) and skeletal muscle oxygenation dynamics (Vernillo et al., 2017) after a mountain ultra-marathon included a control group, this has never been done when measuring changes in the cost of running. The presence of a control group is important, as its inclusion would limit the likelihood of confounding variables (e.g., the selection of the running protocol or the lack of sufficient familiarization) affecting the results.

## Concluding remarks

Studies on RE changes due to ultra-marathons (expressed as O_2_ cost or Cr) report contradictory results. This contrasts with conventional wisdom that Cr typically drifts upwards during or after running exercises up to the marathon distance (e.g., Brueckner et al., 1991). In the present opinion article, we questioned these observed discrepancies, illustrating potential mechanisms associated with a positive or negative effect of fatigue on Cr. Additionally, we discussed the necessity to set up scientific standards to assess Cr changes in ultra-marathon studies. It is our opinion that the design of future studies examining the changes in Cr after an ultra-marathon can be improved by addressing four specific methodological limitations. First, consideration for the specific conditions of the ultra-marathon when designing the running protocol; second, taking into account whether Cr changes between pre- and post-race are consistent across individuals; third, providing adequate familiarization sessions to reduce the effect of habituation; lastly, inserting a control group to reduce biased interpretation of the results.

## Author contributions

GV, GPM, and GYM: Conceived and designed research, analyzed data, prepared figure, drafted manuscript, edited and revised manuscript and approved final version of manuscript.

### Conflict of interest statement

The authors declare that the research was conducted in the absence of any commercial or financial relationships that could be construed as a potential conflict of interest.
